# L-carnitine as a novel approach for pain and inflammation relief in rheumatoid arthritis

**DOI:** 10.1007/s10787-025-01956-y

**Published:** 2025-10-02

**Authors:** Abdallah A. Eldisouky, Sahar K. Hegazy, Salwa Elmorsy Abd Elghany

**Affiliations:** 1 Department of Clinical Pharmacy, Faculty of Pharmacy, Tanta University, Tanta, 315272 Egypt; 2 Department of Rheumatology, Physical Medicine and Rehabilitation, Tanta University, Tanta, Egypt

**Keywords:** L-carnitine, JAK/STAT, TGF-β1, Rheumatoid arthritis

## Abstract

Rheumatoid arthritis (RA) is a chronic autoimmune disorder characterized by joint inflammation, largely mediated by pro-inflammatory cytokines. Considering the established involvement of the Janus kinase/signal transducer and activator of transcription (JAK/STAT) pathway and transforming growth factor-beta1 (TGF-β1) in RA, this research aimed to assess efficacy and safety of L-carnitine as an adjunct therapy targeting these pathways. Forty-six patients with active RA were randomly divided into two equal groups. Group1 (control group) received disease-modifying antirheumatic drugs (DMARDs), including methotrexate, leflunomide, and hydroxychloroquine. Group2 (L-carnitine group) received, DMARDs plus L-carnitine 500 mg twice daily for 12 weeks. A clinical evaluation was conducted, which included tender joint count (TJC), swollen joint count (SJC), pain intensity quantified via the visual analogue scale (VAS), and morning stiffness duration. Additionally, the Disease Activity Score in 28 joints (DAS28) and functional capability as measured by a modified health assessment questionnaire (MHAQ) were assessed. Laboratory evaluation included C-reactive protein (CRP), STAT3, and TGF-β1 measurement. All evaluations were executed both at baseline and following a 12-week treatment period. After 12 weeks, the L-carnitine group showed significant improvement in morning stiffness, VAS, TJC, CRP, DAS28, and MHAQ compared to baseline. While no significant within-group changes were observed in STAT3, TGF-β1 in the L-carnitine group, STAT3 levels increased significantly in the control group compared to baseline. In conclusion, L-carnitine in combination with DMARDs may enhance clinical outcomes in RA by mitigating systemic inflammation. Nevertheless, its impact on STAT3 and TGF-β1 remains unclear and warrants further research.

## Introduction

Rheumatoid arthritis (RA) is a chronic autoimmune disease marked by ongoing synovitis, progressive damage to joint structures, and eventual bone deformities (Sumathi et al. 2022). While synovitis serves as a principal cause of pain in RA, emerging evidence suggests that numerous patients persist in reporting pain despite the apparent control of inflammation (Simon et al. 2021). This persistent pain is attributed to a combination of inflammatory and non-inflammatory mechanisms, with peripheral and central sensitization being implicated as key contributors (Biddle and Sofat 2020).

The JAK/STAT signaling pathway is activated by various cytokines and growth factors, subsequently modulating the expression of genes involved in immune responses, inflammatory processes, and cell proliferation (Morris et al. 2018; O'Shea et al. 2015). Among its components, STAT3 plays a central role by upregulating matrix metalloproteinases (e.g., MMP-2 and MMP-9), stimulating the proliferation of fibroblast-like synoviocytes, and suppressing apoptotic pathways (Ciobanu et al. 2020). Given its central role in the pathogenesis of RA, STAT3 has gained considerable attention as a potential therapeutic target in autoimmune and proliferative disorders (Dutzmann et al. 2015).

TGF-β1 is a cytokine with diverse roles in immune system modulation and tissue remodeling. TGF-β1 levels are significantly elevated in the synovial fluid and tissues of RA patients, where it contributes to disease progression by promoting fibroblast proliferation, synovial hypertrophy, and extracellular matrix degradation (Bira et al. 2005; Cheon et al. 2002). TGF-β1 has also been implicated in the induction of aggrecanase-1, an enzyme responsible for cartilage degradation, thereby exacerbating joint destruction in RA (Yamanishi et al. 2002).

L-carnitine is an endogenous quaternary ammonium compound that enables long-chain fatty acids transport into mitochondria, a process essential for β-oxidation, thus supporting cellular energy metabolism. In addition to its metabolic functions, L-carnitine exhibits antioxidant, anti-inflammatory, and cytoprotective properties by attenuating oxidative stress and influencing key inflammatory pathways (Fathizadeh et al. 2020; Li et al. 2012; Pekala et al. 2011).

Evidences from preclinical studies suggest that L-carnitine may modulate the JAK/STAT signaling cascade, particularly through the suppression of STAT3 expression in inflammatory and tissue injury contexts (Hassan et al. 2021; Mollica et al. 2020). Additionally, several studies have reported that L-carnitine influences the expression and/or activity of TGF-β1, potentially contributing to its regulatory effects in inflammatory processes (Hassan et al. 2021; Salama et al. 2021; Zambrano et al. 2013, 2014).

Considering the roles of the JAK/STAT signaling pathway and TGF-β1 in RA pathogenesis, and given that L-carnitine has been reported to modulate these pathways in other chronic conditions such as cardiovascular, pulmonary, and renal diseases, this study was designed to investigate whether similar effects could be observed in rheumatoid arthritis. We hypothesized that L-carnitine, when administered alongside DMARDs, would reduce disease activity and modulate key molecular markers including STAT3 and TGF-β1.

## Materials and methods

### Study design and ethical approval

This randomized-controlled parallel study comprised 46 patients with active RA, currently receiving conventional DMARDs therapy (methotrexate, leflunomide, and hydroxychloroquine), who were randomly assigned into 2 equal groups. Group 1 (the control group; *n* = 23) was administered DMARDs therapy exclusively, while Group 2 (the L-carnitine group; *n* = 23) received L-carnitine (Carnitol 500mg capsules, Global Napi) as an adjunct to the ongoing DMARDs treatment for a duration of 12 consecutive weeks. Evaluations of the patients were conducted at baseline and at the completion of week 12. The study was approved by the local research ethics committee with approval code (No: 36264MS83/2/23). Additionally, the study was registered with ClinicalTrials.gov under ID: NCT05792527. Written informed consent was obtained from all the participants involved in this research. This study was executed according to the code of ethics of the World Medical Association "Declaration of Helsinki" and the guidelines for Good Clinical Practice.

### Participants

A total of 46 patients with active RA, fulfilling the 2010 RA classification established by "American College of Rheumatology /European League Against Rheumatism" (Aletaha et al. 2010), were recruited. For inclusion eligibility, patients must be administered conventional DMARDs’ therapy for RA on a regular basis, irrespective of sex, and within the age range of 18–70 years. Exclusion criteria encompassed patients with a history of heart disease, renal or hepatic dysfunction, or those currently receiving biological treatment for RA. Additionally, patients taking oral prednisolone exceeding 15 mg/day, those with hypersensitivity to study medications, or those consuming antioxidants were excluded. Furthermore, pregnant and lactating females and patients with a changed drug treatment schedule throughout the duration of the study were also excluded. The pharmacological regimen was maintained consistently throughout the duration of the study for all participants.

### Clinical assessments

Clinical examinations were conducted by a rheumatologist prior to and 12 weeks following the intervention. Disease activity was assessed utilizing the DAS28-CRP, a composite score that incorporates the count of tender and swollen joints, the patient's overall evaluation of disease activity, and the level of CRP. The DAS28 grading system assigns the following categories: remission (< 2.6), low (2.6–3.2), moderate (3.2–5.1), and high (> 5.1). Moreover, the duration of morning stiffness, pain intensity evaluated by the VAS (Haefeli and Elfering 2006), as well as functional capability assessed by the MHAQ (Pincus et al. 2005) were measured.

### Laboratory assessment

#### Blood sample collection and analysis

Prior to the initiation of L-carnitine therapy (baseline) and upon completion of the 12-week intervention period, venous blood specimens (10 mL) were collected from each participant utilizing a disposable sterile plastic syringe. Each specimen was processed as follows: 3 mL were allocated for C-reactive protein (CRP) assessment in the laboratory of Tanta University Hospital employing an immunoturbidimetric methodology (Sişman et al. 2007), while the residual 7 mL were subjected to centrifugation at 3000 rpm for 10 min to isolate serum, which was subsequently stored at -80 °C until analysis. Enzyme-linked immunosorbent assay (ELISA) kits (DLR-Develop, China; Catalogue No. DLR-STAT3-Hu for STAT3 and DLR-TGFb1-Hu for TGF-β1) were utilized to ascertain serum concentrations in adherence to the manufacturer's guidelines.

#### Tests principle

These assays employ a microtiter plate pre-coated with antibodies specific to either STAT3 or TGFβ1. Samples and biotin-conjugated detection antibodies are added, followed by Horseradish Peroxidase-conjugated Avidin. A colorimetric change occurs upon addition of TMB substrate, indicating the presence of the target protein. The reaction is stopped with sulfuric acid, and absorbance is measured at 450 nm to quantify protein levels by reference to a standard curve.

### Primary and secondary outcomes

#### Primary outcomes

The primary efficacy endpoints of the study encompassed the alterations noted in the DAS28-CRP, in conjunction with scores derived from the MHAQ and VAS, assessed from baseline to the conclusion of the 12-week treatment duration.

#### Secondary outcomes

The secondary outcome involved assessing changes in the serum concentrations of the selected biological markers.

### Calculation of sample size

The G*Power software (version 3.1.9.7) was used to determine the sample size, predicated upon an expected moderate-effect size (*f* = 0.25) for detecting intergroup differences in the primary outcome (DAS28-CRP). The significance level (*α*) is established at 0.05, with a statistical power (1 − *β*) of 80%, the estimated sample size was 42 participants. To accommodate a possible 10% dropout rate, the final sample size was increased to 46 patients, randomly allocated in equal numbers to two parallel groups.

### Statistical analysis

Statistical analyses were performed utilizing IBM® SPSS® Statistics, version 28 (IBM Corp., Armonk, NY, USA). The assessment of data normality was carried out via the Shapiro–Wilk test or the Kolmogorov–Smirnov test, as deemed appropriate. Parametric variables were analyzed employing paired and unpaired t tests to compare means within groups and between groups, respectively. The Mann–Whitney U test was applied to non-parametric variables. Results are denoted as mean ± standard deviation (SD), with a *p *value ≤ 0.05 regarded as statistically significant.

## Results

### Patient recruitment

**Fig. 1:**
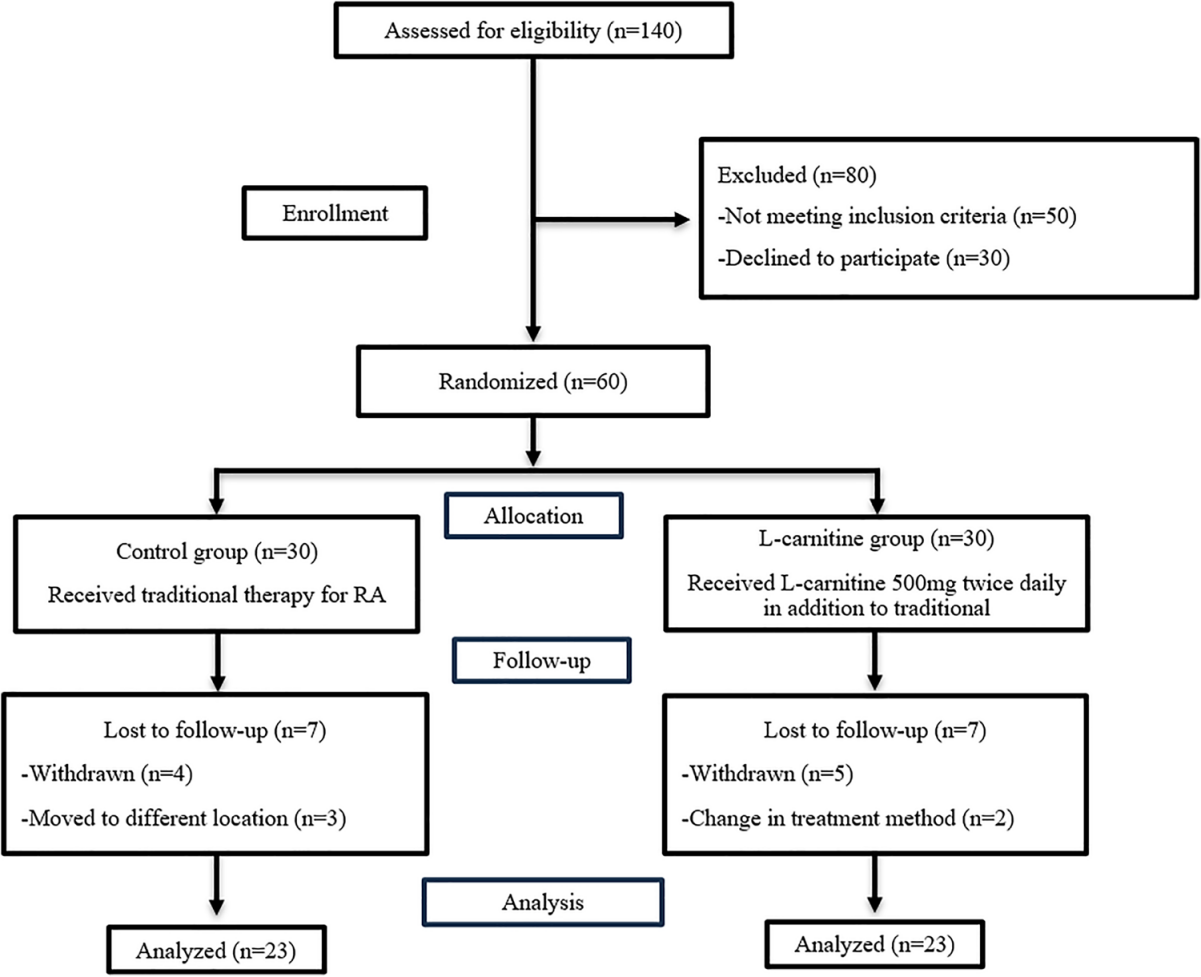
CONSORT flowchart of the study

Participants were recruited from Rheumatology, Physical Medicine, and Rehabilitation Department, Tanta University Hospital, between March 2023 and March 2024. Figure 1 demonstrates the flow of study participants.

### Demographic and clinical characteristics of the study participants

Table 1 provides a summary of the study participants' baseline demographic data. There were no statistically significant differences between Group 1 (control group) and Group 2 (L-carnitine group) in terms of age, sex, weight, or height.

**Table 1:** Baseline demographic characteristics of the participants included in the study

Parameters	Group 1 (control group)	Group 2 (L-carnitine group)
Age (years)	45.5 ± 8.8	48.3 ± 8.2
Gender M: F	2:21	1:22
Weight (kg)	76.7 ± 10.5	79.3 ± 14.9
Height (cm)	162.9 ± 9.1	162.2 ± 9.8
BMI (kg/m^2^)	29 ± 4.3	30.9 ± 5.5

Data are presented as mean ± SD and numbers

BMI: body mass index, M: male, F: female, kg: kilogram, cm: centimeter, m^2^: meter square

Group 1 (control group): 23 patients with active RA received DMARDs therapy for 12 weeks

Group 2 (L-carnitine group): 23 patients with RA received 500mg L-carnitine plus DMARDs for 12 weeks

The clinical and biochemical outcomes for both groups, before and after the 12-week intervention, are demonstrated in Table 2. In the L-carnitine group, there was a statistically significant reduction in morning stiffness, VAS, TJC, CRP levels, DAS28 score, and MHAQ score when compared to baseline (*p* < 0.05). In contrast, no significant changes were observed in STAT3, TGF-β1, or SJC within the same group (*p* = 0.136, *p* = 0.447, and *p* = 0.100, respectively).

**Table 2:** Clinical and biochemical parameters of the participants at baseline and after 12 weeks of treatments in RA patients

Parameters		Group 1 (control group)	Group 2 (L-carnitine group)	*P*^b^ value
		Mean ± SD	Mean ± SD	
CRP (mg/dl)	Baseline	12.13 ± 8.42	24.83 ± 11.57	0.001^*^
	After 12 weeks	21.87 ± 8.29	8.26 ± 6.47	
	*P*^a^ value	< 0.0001^*^	< 0.0001^*^	
STAT3 (ng/ml)	Baseline	0.503 ± 0.27	1.34 ± 1.08	**0.121**
	After 12 weeks	0.704 ± 0.36	0.917 ± 1.07	
	*P*^a^ value	< 0.0001^*^	**0.136**	
TGF-β1 (Pg/ml)	Baseline	1.58 ± 0.22	1.21 ± 0.45	0.032^*^
	After 12 weeks	1.42 ± 0.30	1.39 ± 0.91	
	*P*^a^ value	0.018^*^	**0.447**	
DAS28	Baseline	4.44 ± 0.74	4.86 ± 0.64	0.001^*^
	After 12 weeks	4.71 ± 0.71	3.91 ± 0.79	
	*P*^a^ value	0.020^*^	< 0.0001^*^	
SJC	Baseline	3.87 ± 3.46	2.83 ± 2.66	0.005^*^
	After 12 weeks	4 ± 2.78	1.96 ± 1.61	
	*P*^a^ value	**0.540**	**0.100**	
TJC	Baseline	9.48 ± 5.04	12.17 ± 4.59	**0.337**
	After 12 weeks	9.04 ± 4.71	7.61 ± 5.19	
	*P*^a^ value	**0.306**	0.001^*^	
VAS	Baseline	7.26 ± 1.25	7.96 ± 0.88	< 0.0001^*^
	After 12 weeks	7.74 ± 1.25	5.48 ± 1.24	
	*P*^a^ value	**0.064**	< 0.0001^*^	
Morning stiffness (Minutes)	Baseline	28.04 ± 19.99	32.83 ± 36.14	**0.083**
	After 12 weeks	27.39 ± 18.46	21.52 ± 26.30	
	*P*^a^ value	**0.864**	0.001^*^	
MHAQ	Baseline	1.87 ± 0.69	1.88 ± 0.67	< 0.001^*^
	After 12 weeks	1.82 ± 0.49	1.32 ± 0.59	
	*P*^a^ value	**0.547**	< 0.0001^*^	

Data are expressed as mean ± SD, the significance level was set at *p* < 0.05

CRP: c-reactive protein, STAT3: Signal transducer and activator of transcription 3, TGF-β1: Transforming growth factor-beta1, DAS28: disease activity score, SJC: swollen join count, TJC: tender joint count, VAS: visual analogue scale, MHAQ: modified health assessment questionnaire, mg: milligram, ng: nanogram, pg: picogram, mL: milliliter, dl: deciliter

*P*^a^ value: the probability of significance within the same group (at baseline and after treatment)

*P*^b^ value: the probability of significance between the two groups after 12 weeks of treatment

* Statistically significant

In the control group, there was a statistically significant increase in CRP levels, DAS28 scores, and STAT3 concentrations compared to baseline (*p* < 0.05). Additionally, there was significant decrease in TGF-β1 levels (*p* < 0.05). No other parameters showed statistically significant changes in this group following the 12-week period.

When comparing both groups after 12 weeks of treatment, the L-carnitine group demonstrated significantly lower levels of CRP and TGF-β1, as well as improved DAS28 scores, reduced SJC, pain intensity (VAS), and MHAQ scores (*p* < 0.05). No significant differences were observed in the remaining clinical or biochemical parameters between the two cohorts at the conclusion of the study duration.

### Safety

All participants tolerated L-carnitine well, and no negative side effects reported throughout the study duration. No clinical or laboratory signs of toxicity or intolerance were observed, indicating a favorable safety profile. These results align with prior research that has reliably documented the safety of L-carnitine supplementation across various populations (Malaguarnera 2012; Rebouche 2004).

## Discussion

The present study investigated the therapeutic potential of L-carnitine in mitigating inflammation and improving clinical outcomes in active RA patients, with a particular emphasis on the JAK/STAT signaling pathway and TGF-β1. Administration of L-carnitine as an adjunct to DMARDs therapy over a 12-week period resulted in significant reductions in CRP levels, morning stiffness, pain intensity measured via VAS, TJC, DAS28 score, and MHAQ score. In contrast, participants in the control group exhibited deterioration in several inflammatory and clinical parameters, including a significant increase in CRP levels, and STAT3 and DAS28 scores.

The observed reduction in CRP levels following L-carnitine supplementation supports its anti-inflammatory potential. This result is consistent with the findings of Haghighatdoost et al. (2019), who reported significant decreases in CRP and other pro-inflammatory biomarkers in patients with metabolic and inflammatory disorders receiving L-carnitine supplementation.

Although STAT3 levels did not show a statistically significant reduction in the L-carnitine group, the lack of a notable increase observed in the control group suggests a possible stabilizing or protective effect. This outcome aligns with the findings of Hassan et al. (2021) and Mollica et al. (2020), who demonstrated that L-carnitine mitigated STAT3 activation in experimental models of inflammation.

Concerning TGF-β1, no statistically significant alteration was noted within the L-carnitine group in comparison to baseline measurements; however, post-treatment comparisons between groups revealed a significant difference favoring the L-carnitine group. This finding suggests that L-carnitine may exert modulatory effects on TGF-β1 expression, potentially mediated by its antioxidant and anti-inflammatory properties. Comparable effects have been documented in experimental studies, wherein L-carnitine mitigated tissue damage by modulating signaling pathways linked to TGF-β1 regulation (Salama et al. 2021; Zambrano et al. 2013, 2014). It is possible that, in the context of RA, L-carnitine may impact TGF-β1 levels indirectly or require longer treatment durations to manifest a measurable effect, given the complex regulatory networks involving TGF-β1 in fibrosis and immune responses.

The improvement in clinical parameters including DAS28, TJC, VAS, morning stiffness, and MHAQ in the L-carnitine group further supports its potential as an adjunct therapy. These results align with the observations of Schweiger et al. (2025), who documented significant pain relief and improved physical function in patients receiving L-carnitine.

Nevertheless, the absence of significant changes in swollen joint count (SJC), STAT3, and TGF-β1 within the L-carnitine group may indicate that certain pathological aspects of RA—particularly those involving deeper synovial or structural alterations—might necessitate extended treatment durations or higher dosages to elicit a therapeutic response.

## Conclusion

L-carnitine, when administered alongside conventional DMARDs therapy, demonstrated significant improvements in systemic inflammation and clinical manifestations among patients with RA. Despite inconclusive evidence regarding its direct impact on STAT3 and TGF-β1, the observed positive clinical and biochemical results underscore its potential therapeutic value. Further large-scale, long-term investigations are warranted to validate these findings and to thoroughly assess the full impact of L-carnitine on RA management.

## Limitations of the study

The study enrolled patients with active RA, with a significant proportion receiving dual or triple DMARDs’ therapy. The limited cohort of patients receiving monotherapy restricted the feasibility of executing subgroup analyses to evaluate the interaction between L-carnitine and each individual DMARDs. Further researches, encompassing a larger, stratified, and including newly diagnosed patient population, are requisite to further elucidate these potential interactions.

## Data Availability

Data are available upon reasonable request from the corresponding author.
